# Repair of Block Masonry Panels with CFRP Sheets

**DOI:** 10.3390/ma12152363

**Published:** 2019-07-25

**Authors:** Marco Corradi, Giulio Castori, Romina Sisti, Antonio Borri, Giovanni Luca Pesce

**Affiliations:** 1 Department of Engineering, University of Perugia, 6156 Perugia, Italy; 2 Department of Architecture and Built Environment, University of Northumbria, Newcastle Upon Tyne NE1 8ST, UK

**Keywords:** earthquake engineering, laboratory testing, block masonry, CFRP, shear behavior

## Abstract

In the 1980s, block masonry started to be widely used for new constructions in Italy’s earthquake prone areas. However, recent seismic events demonstrated that block masonry buildings may need to be repaired after earthquakes due to cracking. Construction defects are the main cause for cracking of block work masonry. Carbon fiber reinforced polymer (CFRP) sheets have been used as a local repair method for non-defective and defective wall panels. An experimental program was formulated to investigate the shear behavior of block masonry walls repaired with CFRP sheets. A total of six wall panels were constructed in the laboratory and tested in shear (in-plane lateral loading). It was found that, although the control (non-defective) wall panels had a high ultimate load capacity, the use of CFRPs reduces the effects of construction defects and restores the lateral load capacity in non-defective walls. Overall, this research suggests that the use of epoxy-bonded CFRP sheets could be used for local repair of cracked wall panels.

## 1. Introduction

The use of hollow fired clay (terra cotta) blocks for new constructions is popular in many European countries. It was the extrusion machine invented by the Marquis of Tweeddale in 1836 [1] that simplified the manufacture enough to bring hollow clay into more general use not only for new constructions, but also for enlargement and repair of historic buildings. In many areas of Europe, it is common nowadays to find historic constructions made with the original stonework masonry at the ground floor and with hollow clay blocks on the first and second floor [2,3,4].

There are several reasons for the increase in popularity of hollow clay and concrete blocks in construction including their high compressive strength and durability, high fire resistance, reduced weight and cost. Clay load-bearing hollow blocks are easy to install due to their uniform size and shape. The blocks are compacted by the high pressure of the extrusion, which makes them very strong and able to withstand high vertical loads. Furthermore, one typical clay hollow block may replace up to ten traditional solid bricks, and thereby reduce the construction cost and duration. Their low weight facilitates rapid construction work and the penetration of the mortar in the block’s voids during construction promotes high mechanical interlocking at the block-to-mortar interface, substantially enhancing the structural response of this type of masonry. Load-bearing clay blocks can also improve the energy efficiency of the buildings [5,6,7] as the external envelope is the most important part of any structure with regard to heat loss or heat gain and the thermal conductivity of clay blocks is typically very low, resulting in a significant reduction in heating or cooling costs.

However, the vulnerability of recently constructed masonry buildings to earthquakes, including the hazard from progressive damage, received limited attention by the research community. Buildings surveys of the typical masonry typologies used in Italy and Europe for construction in the 1980s and 1990s are outlined in Magenes and Calvi [8], Lu and Kasa [9], and Mendes et al. [10]. In these studies, it was demonstrated that Italy’s unreinforced masonry (URM) building stock constitutes not only historic masonry buildings, made with rubble and squared stones or solid bricks, but also an increasing percentage of hollow load-bearing block masonry [11]. Due to its high resistance to seismic forces, the Italian Building Codes [12] promoted the use of hollow load-bearing block in URM masonry in most seismic areas. Many block masonry buildings now exist; of these, many are rural residences in areas on the Apennines at a high seismic risk. This masonry typology has also been widely used for reconstruction of portions of historic buildings, often protected by the Italian Regional Conservation Bodies (*Sovrintendenza Archeologia, Belle Arti e Paesaggio*).

New legislation has been introduced in Italy for regulating the construction of buildings made with hollow load-bearing blocks, and strengthening is sometimes needed to meet a required standard, especially to repair damaged buildings. Seismic retrofit is sometimes more expensive than demolition and therefore, the latter may be more attractive to the owners when compared to the costs of reinforcement. However, the choice of the most appropriate intervention often depends on the type and level of damage. Typical mechanical characteristics, crack patterns, and failure modes are critical information for structural engineers involved in the design of retrofitting interventions. 

**Figure 1:**
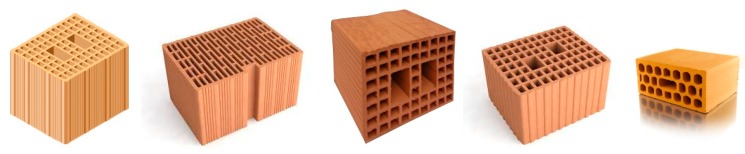
Different types of blocks available on the construction market.

**Figure 2:**
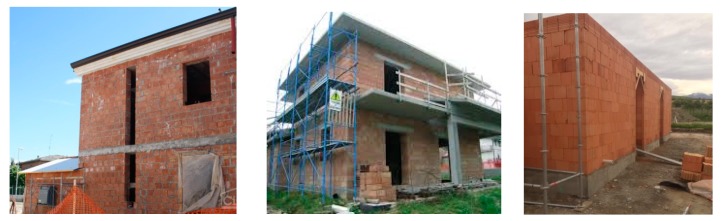
Example of residential block masonry buildings in Italy.

However, little information is available about the failure mechanisms of hollow blocks in URM masonry [13,14,15]. This is mainly because there is limited evidence of damaged buildings, given the relatively recent use of this masonry typology in Italy and in the rest of the world. This is probably because the typical hollow load-bearing blocks used in Italy (Figure 1 and Figure 2) were rarely employed in the US and other seismic areas, where other masonry typologies were commonly used.

## 2. Research Significance

**Figure 3:**
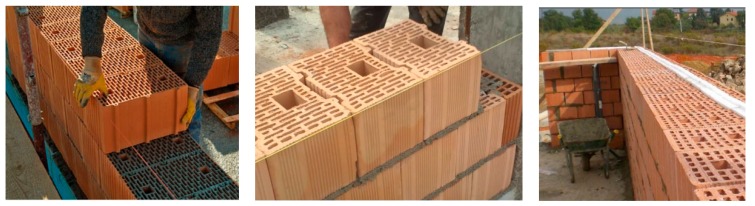
Construction method of hollow clay blocks: blocks are typically arranged in single-wythe walls, without mortar along the vertical joints.

Single-wythe clay block masonry walls provide the entire wall thickness of an exterior building wall (Figure 3). Such single-wythe walls are therefore quite different from historic stonework walls consisting of a multi-leaf wall made of ashlar (or rubble) stones and lime mortar. 

The seismic event in Central Italy in 2016 produced extensive damage to the local building stock [16,17,18] and on many occasions, engineers faced the problem of repairing hollow clay block URM masonry, especially when the reported damage was limited and demolition was not an economically viable solution. In dealing with this task, unfortunately the experts do not have the support of guidelines or building codes. 

This problem may be overcome by using both innovative and conventional methods. Fiber reinforced polymer (FRP) materials have demonstrated to be an effective reinforcement or repair material for masonry structures. However, although extensive research has been conducted for historic masonry structures (wall panels, pillars and columns, vaults and arches), very limited information is available on the applications of this material to more modern hollow block masonry buildings [19,20,21,22,23,24]. FRP sheets, meshes or strips are typically bonded to the surface of structural elements to improve their shear strength or lateral stiffness. The reinforcement of masonry buildings with FRP is particularly cost-effective, as it minimizes disruption of use, and reduces the demolition and reconstruction process of damaged structural members. This composite material provides a favorable strength-to-weight ratio, is non-corrosive, and is easily installed on site using organic or inorganic matrices. 

This research aims to study the most effective methods for the repair and reinforcement of hollow block URM masonry. A preliminary analysis of the damage mechanisms and their validation using laboratory-based tests will be addressed in this paper. While extensive analysis was conducted for historic buildings, much less has been documented for hollow block URM masonry structures [25,26,27,28,29]. Non-defective and defective block masonry panels have been tested in shear, before and after the application of a CFRP (Carbon fiber reinforced polymer) repair. The objective of this paper is to examine the effectiveness of a local application of CFRP sheets to ‘stitch’ a crack in the masonry. Particular emphasis will be placed on the masonry structures struck by the earthquake of 2016 in Central Italy.

## 3. Survey of the Damage after the 2016 Umbrian Earthquake

The use of fired clay hollow block has become very popular in Umbria since the 1980s. However, the structural response of this type of masonry was never really tested by a destructive earthquake before 2016. 

Four major seismic events hit the Nera valley, in Umbria (Italy), between August and September 2016 (Aug. 24 at 3:36 a.m., Oct. 26 at 7:11 p.m. and 9:18 p.m., and Oct. 30 at 7:40 a.m. local time) with a maximum magnitude of 6.5 ML (Richter scale). Residents of Umbria, and nearby regions of Lazio and Marche, felt the earthquakes, which caused 299 causalities and heavy damage to the building stock, especially historic buildings. With regard to URM hollow load-bearing block masonry, the structural response of buildings was significantly better: a post-earthquake survey showed a very limited number of collapses of URM hollow load-bearing block masonry buildings [30]. However, it should be remarked that in most cases these were simple 1-, 2- or 3-story buildings, used as dwelling-houses, stables or dryers. 

The four seismic events heavily struck the area of Norcia, in Umbria where the URM hollow load-bearing block masonry buildings reported a recurrent type of damage: the opening of horizontal cracks in the bed joints. Typically, a single crack caused a horizontal slippage of the overhanging part of the building, with a relative displacement up to 20–30 mm. This is detailed in the following paragraphs where the damage of 3 URM hollow load-bearing block masonry buildings located in the hamlet of San Pellegrino near Norcia, are described.

**Figure 4:**
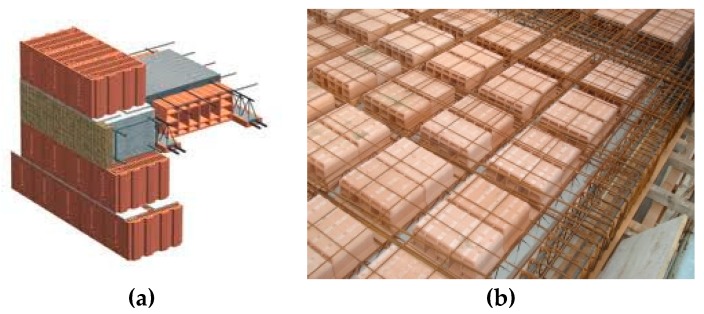
(**a**) Detail of the area near the RC ring-beam, (**b**) a horizontal diaphragm and ring beams ready for casting.

The typical construction method of hollow clay block masonry in Italy consists in the use of 25–40 cm thick blocks, to form a single-wythe wall, and a cement mortar. In order to reciprocally connect the walls, and prevent out-of-plane collapse mechanisms, the Italian Building Code requires the construction of ring beams at each floor level and at the eaves level. These are typically made of a reinforced concrete (RC) [31]. The horizontal and roof diaphragms are usually made of 1-way steel-reinforced concrete joists, tile hollow blocks and a 4–5 cm-thick slab reinforced with steel-wire mesh. The joists are fixed to the ring beams and both the ring beam and the slab are cast simultaneously (Figure 4). This construction method was very common in Umbria in the area struck by the 2016 earthquake. 

### 3.1. Building No.1

Building No. 1 is a 3-story dwelling-house with a horizontal plan of 10.39 m × 7.11 m. The building was constructed in 2006, and the design complied with the 1996 Italian Building Code [32]. The standard requirements at that time were almost the same as the more recent Italian Building Code (2018) [33]. The thickness of the URM hollow load-bearing blocks are 60, 45 and 30 cm, for the first, second and third floor, respectively. The horizontal and roof diaphragms were made of 1-way steel-reinforced concrete joists, tile hollow blocks and a 5 cm-thick RC slab.

**Figure 5:**
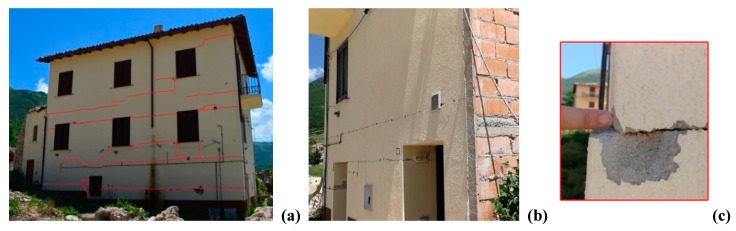
Crack pattern (red lines) of Building No. 1. (**a**) Horizontal cracks from the north; (**b**) horizontal cracks from the east, near the floor and (**c**) detail of the horizontal sliding.

The earthquake in 2016 caused various damage to this building. Figure 5 shows the crack pattern where horizontal cracks opened along the mortar bed joints of the URM hollow load-bearing block masonry. These cracks were mainly concentrated near the joint block masonry—ring beam—floor. The main cause for this is likely to be the stress concentration, induced by the inertial seismic forces, transmitted by the horizontal diaphragms. These are typically very heavy (the dead load alone is about 9–11 kN/m^2^). 

Figure 5b shows detail of the horizontal sliding (20 mm) at the ground floor of the building. Relative displacements are smaller near the 1st and 2nd level joints. It is worth noting that this crack pattern has never been observed for this particular masonry type. 

### 3.2. Building No. 2

**Figure 6:**
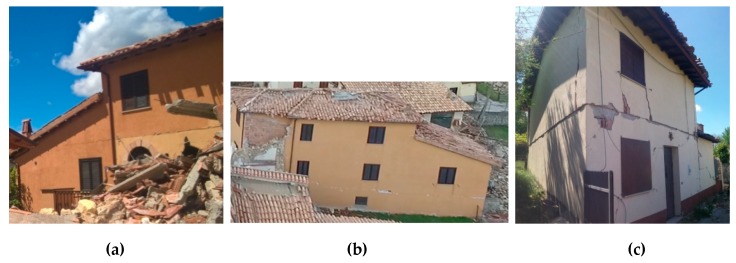
Crack pattern: (**a**,**b**) Building No. 2; (**c**) Building No. 3.

The second construction is a 3-story building. This building has external dimensions of 17.55 m × 4.8 m and it was reinforced in 1986 by demolishing several stonework walls. These were re-constructed with hollow blocks. The only un-demolished pre-exiting stone wall was the one shared with the adjacent building (that was reinforced by grout injections and steel-mesh reinforced concrete coating. The thicknesses of the new URM block masonry are 45 and 30 cm, for the ground floor and the higher levels, respectively. The horizontal diagrams (likely timber-beam floor) were also demolished in 1986 and replaced with 1-way steel-reinforced concrete joists, tile hollow blocks and a RC slab. An indicative configuration of the new floor is shown in Figure 4. During the earthquake in 2016, the building was seriously damaged. A long horizontal crack, passing through the wall thickness, opened near the first level floor (Figure 6a,b). Other horizontal cracks were noted near the floor at ground level. It is worth noting that the typical diagonal cracks, induced by in-plane lateral loading, or the also-common out-of-plane mechanisms, were not observed at all.

### 3.3. Building No. 3

A 2-story building is the last case-study. This building, has a rectangular floor plan of 6.8 m × 9.7 m and was reconstructed in 1989 using new URM load-bearing block masonry. The horizontal diaphragms were made of traditional 1-way steel-reinforced concrete joists, tile hollow blocks and a RC slab. Ring RC beams were also used to connect the floor to the walls to prevent an out-of-plane collapse mechanism during an earthquake (as represented in Figure 4). The 2016 post-earthquake report of the state of the building (“Aedes” report [34]) highlights a medium level of damage. However, the building was evacuated and its use was not authorized. Figure 6c shows the damage: a horizontal crack opened in the external walls at the level of the first floor. This crack passed through the wall thickness. A horizontal sliding of 2 cm of the upper part of the building was measured by the technicians after the earthquake. 

## 4. Numerical Analysis

Despite URM hollow block masonry being very popular in Italy since the 1980s, its structural response has never really been tested by a destructive earthquake before 2016. This is the background of the present study, which aimed at providing relevant data and at numerically investigating the causes of the crack pattern mode of this type of block masonry by means of a commercially available Finite Element (FE) modelling code [35].

**Figure 7:**
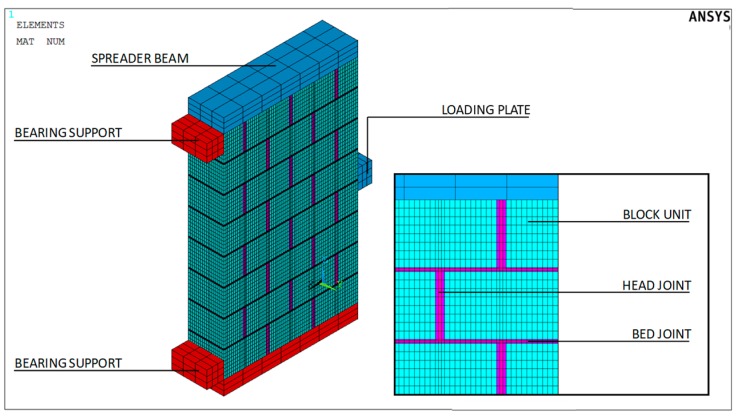
FE model with mesh discretization.

To simulate the behavior of block masonry, a three-dimensional non-linear model was developed using a damage mechanic approach (Figure 7). After performing a sensitivity analysis using different mesh sizes, the FE mesh was refined so as to have eight elements (14 mm × 14 mm × 22.5 mm) across each block unit, three elements (14 mm × 14 mm × 3.33 mm) across each bed joint and three elements (14 mm × 3 mm × 22.5 mm) across each head joint. This guarantees that the more critical details are captured without distorted meshes and, consequently, localization and shear lock effects. Figure 7 illustrates the full FEM (Finite Element Method): it consists of 126,615 elements and 124,632 nodes, with 373,896 DOF.

In such a context, a maximum tensile stress (tensile cut-off) failure criterion was assumed for every masonry component (mortar and block units). Such an elastic-plastic model, originally adopted for concrete and other brittle materials, is able to account for both cracking and crushing failure modes through the use of a smeared model. In detail, the irreversible damage that occurs during the cracking process of both mortar and block units was simulated by using only two material parameters: uniaxial tensile (f_t_) and compressive (f_c_) strength. Furthermore, to improve the reliability of the proposed FE approach, the contact surface between the masonry wall and the bearing supports and load plates, respectively, was modeled through the use of unilateral contact interfaces. In this application, surface-to-surface contact elements were chosen and the contacting properties for the normal and tangent behavior were specified indirectly by a trial-and-error procedure in the calibration process. Specifically, as for the behavior in the tangential direction, a Coulomb friction law was applied to each interface assuming that sliding may (or may not) occur by introducing a friction coefficient (μ = 0.4). The same Coulomb friction contact behavior was used in the normal direction to indicate how a gap can appear when the compressive stresses become negligible.

**Figure 8:**
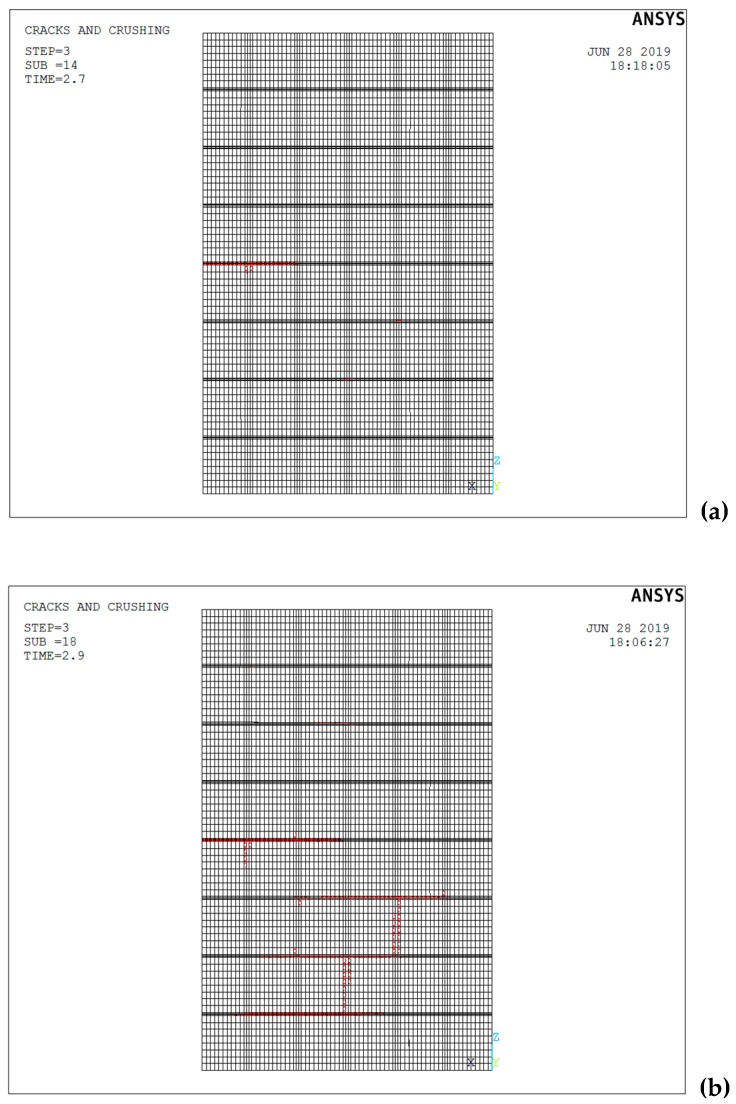
FEM crack pattern: (**a**) opening of the horizontal crack; (**b**) stepped diagonal cracks.

A numerical analysis was thus performed, in which the FE model was firstly subjected to both self-weight and a distributed pressure load, followed by a ramped 10 kN horizontal load. Figure 8 shows the failure progression sequence observed during the FE analysis on the block masonry panel. Cracking is not present on the whole specimen, but mainly on its lower half. In detail, following the opening of a predominant horizontal crack at the panel mid-height (Figure 8a), stepped diagonal cracks developed (through bed and head joints) along the compressed diagonals (Figure 8b), when the interface bond strength was attained.

## 5. Test Program

### 5.1. Specimen Description

**Figure 9:**
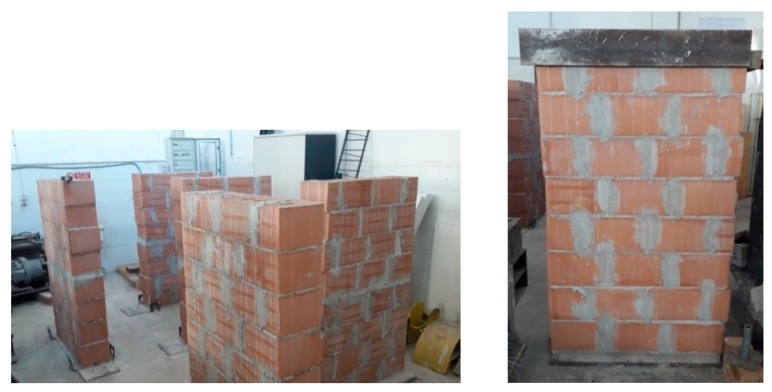
Laboratory testing: the wall panels.

Six wall panels of 1.60 m × 0.90 m × 0.25 m were constructed in the Structures Laboratory at the University of Perugia (Figure 9). These were assembled using hollow load-bearing clay blocks and a ready-to-use cement mortar. The panels were made of 8 courses of blocks: the first course in all specimens was laid with three full-length brock units. Half-length units at each end were used for the subsequent course. The pattern was repeated three more times for the subsequent courses. A running bonding pattern was used and the walls were one-block-thick (single-wythe).

### 5.2. Construction Materials

**Figure 10:**
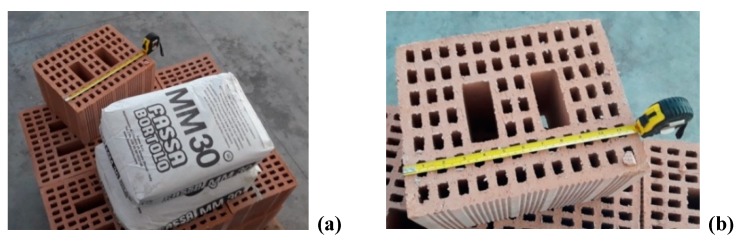
(**a**) Ready-to-use cement mortar, (**b**) 300 mm × 250 mm × 180 mm hollow fired clay block.

Materials used for building the wall panels were tested individually to determine their mechanical characteristics. Vertically perforated fired clay blocks of 300 mm × 250 mm × 180 mm (length × width × height, respectively; Figure 10 were tested in compression to failure under non-eccentric load. Blocks were produced by FBM, Dunarobba, Italy [36]. Mean compressive strength was 6.58 MPa, with an average weight of 12.51 kg/block. Table 1 shows the main results of the mechanical properties of the fired clay blocks and cement mortar.

The mortar used was a ready-to-use MM30 Fassa Bartolo, containing Portland cement, lime and sand (the same used to construct the walls). Mortar prisms of 40 mm × 40 mm × 160 mm were tested in bending according to the EN 1015-11 standard [37]. Once the bending tests was performed, each remaining half of the prisms was tested for compression considering a loading area of 40 mm × 40 mm. Results of the bending and compressive tests are reported in Table 1. 

### 5.3. CFRP

**Figure 11:**
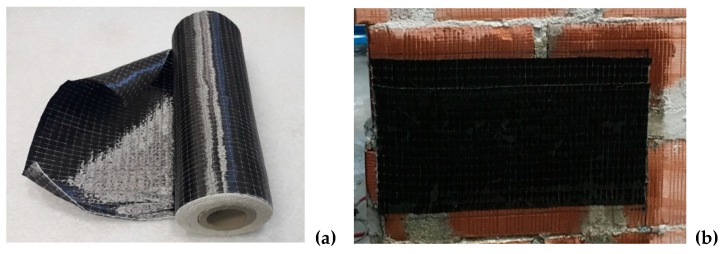
(**a**) Uni-directional CFRP sheet, used for local repair, (**b**) detail of a repaired wall panel.

To repair the wall panels, only one type of CFRP composite was used: this was made from carbon fibers embedded in an epoxy resin matrix to form a unidirectional CFRP sheet, as shown in Figure 11. The 0.165 mm thick CFRP sheet had a tensile strength of 3,324 MPa with a tensile modulus of 312.2 GPa (Table 2). The rupture strain was 1.07%. The same epoxy resin employed to cure the carbon fibers was also used to apply the fibers to the wall’s surface. The epoxy resin is produced by Kimia, under the brand name Kimitech-ep-in: this is a low-viscosity, transparent, bi-component product. The manufacturer declares in the data sheet a compressive strength of 65 MPa, and a tensile strength of 30.4 MPa. The weight density of the epoxy resin is 1.08 g/cm^3^. The surface of the wall panels was not treated to improve the bond performance: the CFRP was directly glued to the tile blocks. 

### 5.4. Test Arrangement

**Figure 12:**
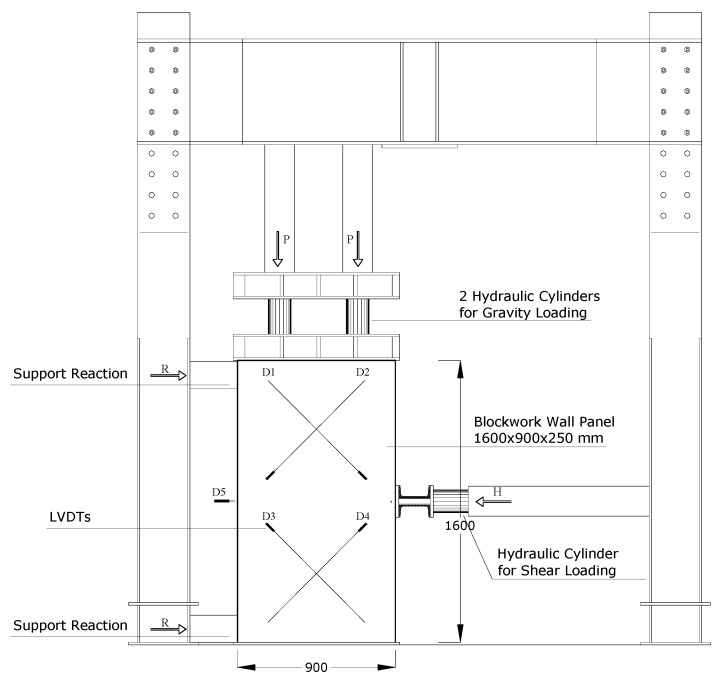
Layout of the shear test (units in mm).

**Figure 13:**
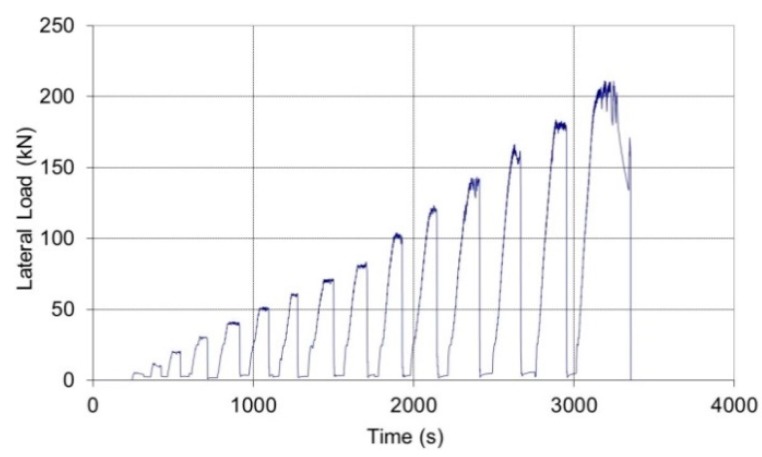
Typical load history: loading and unloading cycles were increased by 10 kN per cycle up to 70 kN, the increment was 20 kN after that. The horizontal lateral load H acted on the wall for a duration of 30 s, and, the panel was subsequently left unloaded for a further 30 s.

Full-scale masonry panels (a total of six wall panels were tested: two non-defective and four defective panels) were built at the laboratory and in-plane tested using the shear-compression test method. An MTS (Eden Prairie, MN, USA) steel load frame was used for testing (Figure 12). Panels were simultaneously subjected to a vertical, almost constant, compressive stress of 0.2–0.3 MPa (normal to the bed joint and needed to simulate the gravity loads of two or three additional floors) and a cycling and increasing horizontal shear load up to failure. Single-acting 50 ton hydraulic cylinders were used for the application of the vertical loads (loads P in Figure 12). The oil pressure in the jacks (vertical force) remained almost constant at the default value until the formation of the first cracks in the wall panel. A rigid deep steel beam was used to uniformly distribute the vertical load on the horizontal section of the panels. The deep beam was placed atop a 1 cm-thick mortar bed. A hydraulic piston was placed along the horizontal line of symmetry (midpoint): this served for the application of the shear in-plane load (load H in Figure 12). The load was manually applied at the rate of about 0.4–0.6 kN/s (Figure 13). The forces (both vertical P and horizontal H) were measured using a pressure gages located near the manual pumps.

Each wall panel was constructed on a reinforced concrete foundation. The test configuration can be efficiently described using the scheme of a three-point bending test on a vertical deep beam. The two end-supports were made of timber prisms (300 mm × 100 mm × 100 mm) and two steel plates (200 mm × 100 mm) were used for a better distribution of the constraint reactions and for preventing local failures. In the analysis of the results, the 1.6 m × 0.9 m blockwork panel was considered as two adjacent, overlapping 0.8 × 0.9 semi-panels (half-panels). Given the symmetry (in terms of geometry, materials and loading conditions), the shear load was equally divided between the two semi-panels. Four LVDTs (Linear Variable Differential Transformers with a measuring range of 50 mm, produced by HBM (Hottinger Baldwin Messtechnik GmbH, Darmstadt, Germany) were used to measure the diagonal deformations of both semi-panels. A further three transducers were placed near the panel’s horizontal line of symmetry to record the horizontal movements and the vertical movements near the loading cylinders.

### 5.5. Test Results

#### 5.5.1. Control Non-Defective Walls

**Figure 14:**
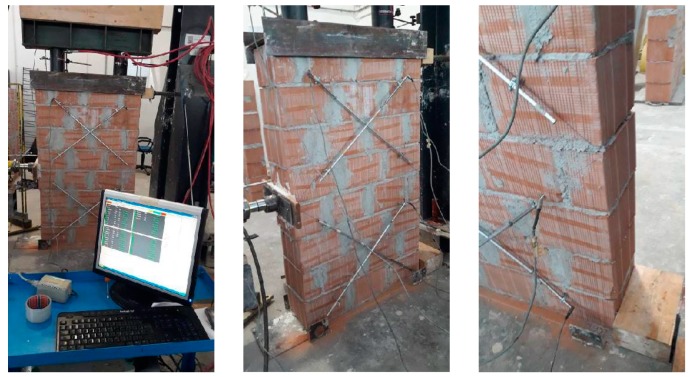
Non-defective wall panels.

Following the initial application of the vertical load, up to the limit of 45 or 67.5 kN (corresponding to a compressive stress of 0.2 or 0.3 MPa, respectively), the shear horizontal load was applied in cycles of increased magnitude, up to failure (Figure 14). The overall structural response of the walls was very satisfactory, with high lateral load capacities varying between 150.55 and 182.98 kN, corresponding to a shear strength of 0.2493–0.3187 MPa. If these values are compared with the shear strength of other types of masonry (solid bricks, stonework, etc.), the hollow block masonry results are much stronger [39,40]. As soon as the principal tensile stress reaches the tensile strength of the cement mortar, a crack forms and failure occurs.

**Figure 15:**
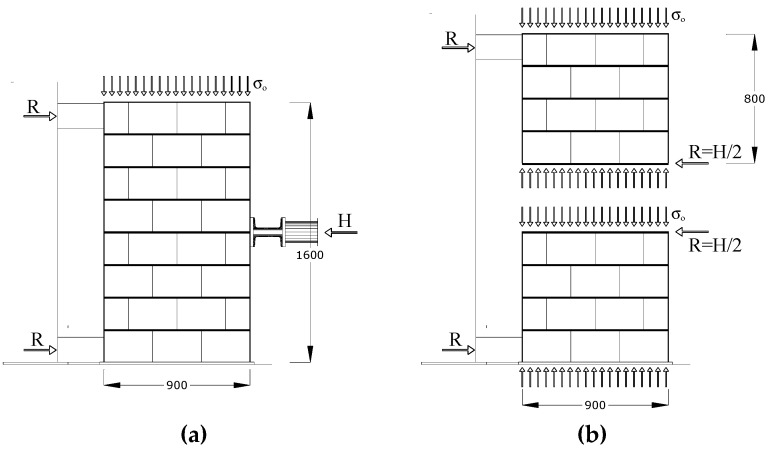
(**a**) Test layout, (**b**) method of calculation of the shear load (R) and strength (τ_0_) by dividing the panel in two halves (semi-panels). Equilibrium of moments of the two semi-panels is achieved by considering the bending moment (M = R × 800 mm) acting along the horizontal line of symmetry of the wall panel on both semi-panels (units in mm).

Each 1600 mm × 900 mm × 250 mm wall panel can be studied by considering the two 800 mm x 900 mm × 350 mm halves (Figure 15). The cracks only opened in the vertical joints and in the horizontal mortar joints, following a “zig-zag” pattern: the vertically perforated hollow blocks were undamaged or only barely damaged after testing. In order to evaluate the shear strength τ_0_ of the masonry, the well-known Turnšek and Cacovic formulation [41] was used as reported in Equation (1):

(1) $$\tau_{0}=\frac{f_{t}}{1.5}$$

where *f_t_* represents the tensile strength of the masonry, given by:

(2) $$\frac{R}{Dt}=\frac{f_{t}}{b}\sqrt{1+\frac{\sigma_{0}}{f_{t}}}$$

where *R* is 50% of the maximum shear load [*R = H/2*] (assuming an equal distribution of the lateral load between the two halves of the wall panel), σ_0_ is the vertical compressive stress (0.2 or 0.3 MPa), *t* is the panel thickness, and *b* is a parameter dependent on the panel aspect ratio *H/D* (*H* = height of the half-panel, *D* = width of the half-panel) and accounts for the distribution of shear stress. This was assumed to be equal to 1.

**Figure 16:**
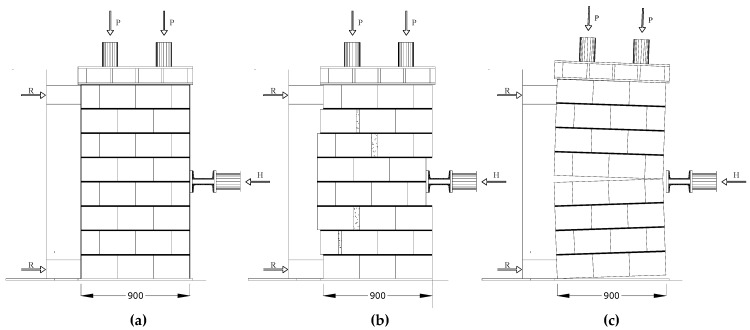
(**a**) Un-deformed wall panel, (**b**) shear failure (diagonal cracking), (**c**) bending failure (horizontal cracking) (units in mm).

The test results seem to confirm the on-site post-earthquake survey of damaged buildings (Table 3). The non-defective panels exhibited a positive seismic response: horizontal failure loads were 158.68 and 150.55 kN, for a vertical stress of 0.2 and 0.3 MPa, respectively. The mean shear strength, calculated with Equation (1), was 0.156 MPa. Two different failure modes were recorded for non-defective panels: shear failure (diagonal cracking—Figure 16a,b) and local crushing in the area near the application of the horizontal lateral load. It should also be noted that test result for P1-ND-30 represents a lower bound value of the shear capacity: without the local crushing, the lateral capacity would be higher than 150.55 kN. 

**Figure 17:**
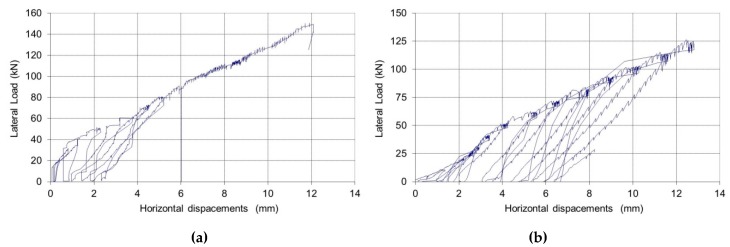
(**a**) Lateral load vs. horizontal displacement for non-defective (P1-ND-30), and (**b**) for the defective panel (P5-DE-20).

The panels’ response in terms of deformations (horizontal displacements of the LVDT D5 along the panel’s line of symmetry, exhibited a linear lateral load-horizontal displacement relationship for low horizontal loads, turning un-linear near the failure load. Figure 17a shows the lateral load vs. horizontal displacement for the non-defective (P1-ND-30).

**Figure 18:**
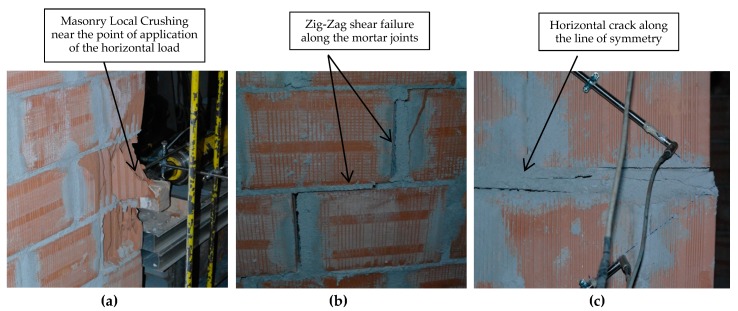
Failure modes: (**a**) un-defective control panel: local crushing; (**b**) un-defective control panel: diagonal cracking (zig-zag pattern), (**c**) defective control panel: horizontal crack along the panel’s horizontal line of symmetry.

The results demonstrated that non-defective panels exhibited a very high shear strength: the average value was 0.156 MPa. This included the result of the P1-ND-30 sample, where local crushing was recorded (Figure 18a). If this result is excluded, the shear strength of non-defective panels was 0.175 MPa. The reader should be alerted about the limited number of available test results. Few standards provide information about mechanical properties of different masonry typologies to be used for design and calculations. The recent Italian Guidelines [42] provide such data. The guidelines suggests the range value of 0.08–0.17 MPa for hollow tile block masonry with cement mortar. It is interesting to note that our results demonstrate that the tested block masonry exhibited a much higher shear strength. Obviously, the Code provides characteristic values, including safety factors, but it could be suggested that the Code underestimates the mechanical properties of block masonry.

#### 5.5.2. Control Defective Walls

The defect was introduced by altering the construction of the wall panel that was assembled in two stages. At the end of the construction of the bottom half (0.9 m × 0.8 m × 0.25 m), a layer of mortar was laid over the panel. Following a two-week break, the top half panel was added, starting from the application of a new layer of fresh mortar over the previous hardened one. This assembly was chosen to simulate a real situation: the construction works of the walls are typically interrupted when the first level is completed, as it is necessary to add, first, the horizontal diaphragm (floor). Subsequently, the construction of the walls continue for another level.

The failure mode of the defective panels entailed a different mechanism, compared to the non-defective ones (Figure 16c and Figure 18c). Two limit states were noted for defective wall panels: a horizontal crack progressively opened between the two overhanging semi-panels (along the wall’s horizontal line of symmetry). The mechanism consisted in a relative rotation around the point of application of the horizontal load of the two halves. This had a maximum thickness of 8–12 mm at the maximum horizontal load. The vertical confinement of the panel prevented further separation and rotation of the two halves. By comparing the maximum lateral load, it can be noted that the defective panels exhibited a reduced shear-load capacity: this was 40.8% smaller compared to non-defective panels. Following the formation of the horizontal crack, shear cracks also developed for a lateral load of 152.89 (P3-ND-20) and 182.98 kN (P4-DE-30) (second limit state), associated with a compressive stress of 0.2 and 0.3 MPa, respectively. These values are consistent with the failure loads recorded for non-defective wall panels (158.68 and >150.55 kN). The mean shear strength was 0.173 MPa.

Regarding the deformation capacity, shear strains (calculated using the shortenings/elongations of the panels’ diagonals) were negligible up to failure (i.e., defective panels did not highly deform in shear) [43]. The bending mechanism was predominant and large horizontal displacements were recorded (LVDT D5 in Figure 12). Horizontal displacements reached 4–7 mm at failure (Figure 17b). Residual deformations (at the end of each loading and unloading cycle) were small as a result of the inverse relative rotation of the two panel’s halves. 

#### 5.5.3. Repaired Defective Walls

All defective panels initially failed due to the formation of a horizontal crack in the bed joint between the two semi-panels. The defect was not critical, and it did not cause a high reduction of the lateral load capacity compared to the control panels: for some panels it was possible to continue to test up to the shear failure. In order to prevent the bending failure mode (horizontal cracking), a double layer of unidirectional CFRP sheets was applied on both sides of the panel. The CFRP repair was made using two 300 mm × 500 mm overlapping sheets, applied on both sides, with the fibers perpendicular to the horizontal crack. After the CFRP repair, defective panels were re-tested in shear, according to the same procedure used to test control panels.

The results of shear tests are summarized in Table 3. It can be noted that the repair was able to prevent the re-opening of the horizontal crack, and restore the original lateral load capacity of the control non-defective panels. The average shear strength τ_0_ of repaired defective panels was 0.2005 MPa (Test No. P4-RE-30 and P6-RE-20). Similar to non-defective wall panels, a single limit state load was recorded for repaired panels. By comparing the first limit state load (Test No. P2-DE-20 and P5-DE-20, Horizontal Cracking Load = 70.08 kN, σ_0_ = 0.2 MPa) of the defective panels, with the limit state load of repaired wall panels (211.28 kN), we note a significant increment in lateral capacity. 

At the beginning of the shear test, the horizontal crack immediately re-opened (up to a thickness of 0.3–0.8 mm), but the activation of the CFRP repair prevented its widening. The structural behavior of both the carbon fibers and the epoxy resin was excellent. The carbon fibers fully absorbed the tensile stresses across the horizontal crack, and the epoxy resin guaranteed an adequate stress transfer between the block masonry and the CFRP sheets. 

**Figure 19:**
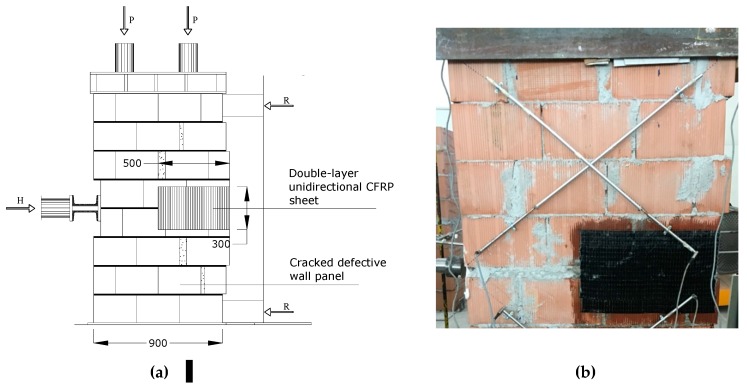
(**a**) CFRP repair, (**b**) typical failure mode of repaired wall panels (detail of upper half panel) (units in mm).

**Figure 20:**
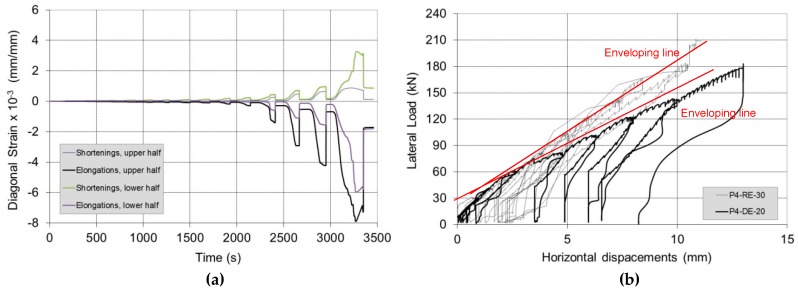
(**a**) Diagonal strains for Test No. P4-RE-30, (**b**) Panel No. 4, before (up to horizontal cracking) and after repair: lateral load vs. horizontal displacements.

By increasing the in-plane lateral load (H), the wall panel started to exhibit increasing shear strains, reaching failure due to diagonal cracking (Figure 19), in one or both of the semi-panels. The failure mode was similar to that observed for non-defective control wall panels: cracks alternately opened in the vertical joints and in the horizontal mortar beds following a “zig-zag” pattern, leaving the tile blocks for the most part un-damaged. Apart from the initial bending deformation (due to the partial re-opening of the horizontal crack), the CFRP repair reversed the effect of the construction defect. Figure 20a shows the development of the angular strains of the two semi-panels during shear loading (Test No. P6-RE-30): the different values of the strains demonstrate that an equal distribution of the shear load between the two semi-panels is likely too simplistic. 

It is worth noting that deboning phenomena or tensile ruptures of CFRP were not recorded in any test. Further tests are necessary to confirm these encouraging results, in particular using different test configurations, sample dimensions, and type of block masonry. However, the emerging line seems quite clear: epoxy-bonded CFRP sheets could be used for local repair of hollow block work masonry.

Figure 20b compares the structural response of Panel No. 4 before and after repair. It can be noted how the application of the CFRP sheet caused an increase in the lateral capacity (although the vertical compressive stress was 0.2 MPa for the P4-DE-20 test and 0.3 MPa for the P4-RE-30 test). In terms of lateral stiffness, it is worth noting that the slopes of the enveloping lines of the curves are very similar. The CFRP repair did not change the shear stiffness of the panel, but only acted to prevent the re-opening of the bending horizontal crack.

## 6. Conclusions

Block masonry wall construction has experienced considerable changes in the 1980s and 1990s with the development of progressively larger and stronger hollow terra cotta blocks and new types of units. The main reasons underlying these changes has been the need for improved thermal insulation, seismic response and speed of construction. 

However, despite the relatively widespread use of hollow load-bearing block masonry, it appears that limited data are available on its seismic behavior. The 2016 Central Italy earthquake damaged a large number of block masonry buildings. Failures were rare, but new crack patterns, previously not listed in the scientific literature, were observed. This paper analyzes such damages and reports the results of an experimental investigation carried out in the laboratory with the aim of studying repair methods using CFRP sheets. A total of eight shear tests were conducted on block masonry panels. It was demonstrated that local repair using CFRP sheets may prevent the bending failure of block masonry wall panels. However, it is difficult to state if a single or a double layer or more is sufficient to prevent the failure mode observed on-site after the earthquake: this depends on the mass of the overhanging parts of the building, the ratio between the stiffness of the structural members, the magnitude of the seismic acceleration and the dimensions of the walls. More tests and analysis will be necessary to address this point. The wall panels were subjected to shear loading. The results suggest the following conclusions:

1. The tests reported herein provide some relevant data on the seismic response of hollow load-bearing block masonry. The test results are of interest because they seem to confirm the on-site evidence of the seismic damage produced by sliding phenomena between the block masonry and the RC beams.
2. The application of a double layer of CFRP sheets was effective in repairing defective cracked panels. The CFRP repair was able to bring the lateral load capacity to the level of the control non-defective panels. It was demonstrated that a CFRP sheet height of 300 mm (150 mm bonding lengths on both semi-panels) is sufficient to prevent detachment or peeling phenomena during the shear test.
3. The use of an epoxy adhesive seems to be critical and fundamental in order to prevent the opening of any further horizontal cracks in the defective panels. The stress concentration in the CFRP is very high, and only a strong bonding agent can be successful in transferring the tensile forces from the masonry material to the carbon fibers.
4. The long-term behavior of the epoxy and the CFRP needs to be further investigated and controlled. Chemical and mechanical degradation of the resin could be a problem in the long run, as well as exposure to high temperatures during the hot summer days. However, degradation could be considered tolerable given the very high initial mechanical properties of both carbon fibers and epoxies.

## Figures and Tables

**Table 1:** Results of material characterization tests.

Property	Fired Clay Hollow Blocks	Mortar
Number of Tested Samples	6	12
Sample Dimensions (mm)	300 × 250 × 180 *	40 × 40 × 80 **
Weight Single Unit (kg)	12.511	0.525
Voids (%)	45 ^+^	-
Failure Load (kN)	475.7	24.65
Compressive Strength (MPa)	6.58 ^++^	15.7
CoV Compressive Strength (%)	11.2	5.79
Number of Tested Samples	-	6
Sample Dimensions (mm)	-	40 × 40 × 160
Bending Strength (MPa)	-	3.75
CoV Bending Strength (%)	-	8.53

* nominal dimensions, ** remaining half of the mortar specimens, after bending test, ^+^ Producer Data Sheet, ^++^ Sectional area inclusive of voids area, CoV = Coefficient of Variation.

**Table 2:** Mechanical properties of the CFRP sheet [38].

Type of Fibres	Carbon
Number of Tested Samples	10
Dry Fiber Thickness (mm)	0.165
Fiber Density (g/m^2^)	300
Matrix Type	epoxy
Tensile Strength (MPa) and (CoV) (%)	3324 (18.1)
Young’s modulus (GPa) and (CoV) (%)	312.2 (19.2)

**Table 3:** Results of shear tests.

Test No.	Vertical Compressive Stress σ_0_ (MPa)	Horizontal Cracking Load (kN)	Shear Failure Load H (kN)	Shear Strength τ_0_ (MPa)	Failure Mode
P1-ND-30	0.3	-	>150.55	0.137	Local crushing
P2-DE-20	0.2	61.71	152.89	0.167	Diagonal cracking
P3-ND-20	0.2	-	158.68	0.175	Diagonal cracking
P4-DE-30	0.3	134.07	182.98	0.179	Diagonal cracking
P4-RE-30	0.3	-	211.28	0.219	Diagonal cracking
P5-DE-20	0.2	78.47	-	-	Horizontal cracking
P6-DE-20	0.2	116.97	-	-	Horizontal cracking
P6-RE-20	0.2	-	167.32	0.182	Diagonal cracking
mean ND-20	0.2	-	158.68	0.156	
mean ND-30	0.3	-	>150.55		
mean DE-20	0.2	85.71	152.89	0.173	
mean DE-30	0.3	134.07	182.98		
mean RE-20	0.2	-	167.32	0.182	
mean RE-30	0.3	-	211.28	0.219	
